# Effects of Stocking Density on the Survival, Growth, and Stress Levels of the Juvenile Lined Seahorse (*Hippocampus erectus*) in Recirculating Aquaculture Systems

**DOI:** 10.3390/biology13100807

**Published:** 2024-10-10

**Authors:** Tingting Lin, Siping Li, Dong Zhang, Xin Liu, Yuanhao Ren

**Affiliations:** 1 Key Laboratory of Inland Saline-Alkaline Aquaculture, Ministry of Agriculture and Rural Affairs, East China Sea Fisheries Research Institute, Chinese Academy of Fishery Sciences, Shanghai 200090, China; lintt@ecsf.ac.cn (T.L.); lisiping@ecsf.ac.cn (S.L.); zhangdong@ecsf.ac.cn (D.Z.); renyh@ecsf.ac.cn (Y.R.); 2 Wenchang Innovation Research Center, Fengjiawan Modern Fishery Industry Park, Wenchang 571300, China

**Keywords:** stocking density, recirculating aquaculture system, seahorse *Hippocampus erectus*, size heterogeneity

## Abstract

**Simple Summary:**

Recirculating aquaculture systems (RASs) have gradually become a highly recommended aquaculture mode due to their environmental friendliness. However, due to their high factory and equipment investments and high electricity consumption, they usually require the farming objects to have high economic value in order to cover their high running costs. Seahorses have high economic value and may be suitable for RASs. Currently, there are almost no reports on the large-scale farming of seahorses using RASs. To provide a new farming object for RASs, in the present study, the lined seahorse (*Hippocampus erectus*) was employed to determine a suitable stocking density in RASs. As is well known, optimizing the stocking density of farmed objects is a prerequisite for their commercial aquaculture. The present study determined suitable stocking densities for lined seahorse juveniles at different growth stages, which will contribute to the development of recirculating aquaculture for the lined seahorse.

**Abstract:**

Seahorses are increasingly regarded as a promising farming object suitable for recirculating aquaculture systems (RASs) due to their high economic value. However, reports on the large-scale farming of seahorses in RASs are rare, and some key parameters, such as stocking densities, are still unclear. In the present study, we employed the lined seahorse (*Hippocampus erectus*), for which large-scale farming has been achieved, to determine the suitable stocking density for three different-sized juveniles in RASs. The three different-sized juveniles had body heights of 4.0, 7.0, and 9.0 cm, and their test density gradients were 1.0, 0.8, 0.6, and 0.4 inds/L; 0.6, 0.5, 0.4, and 0.3 inds/L; and 0.4, 0.3, 0.2, and 0.1 inds/L, respectively. The juveniles were cultivated for one month, and then their survival, growth, and plasma cortisol and brain serotonin contents (two stress-related indicators) were analyzed. The results show that, regardless of the size of the juveniles, a high density can inhibit growth and trigger stress responses. In addition, for small- (4.0 cm) and medium-sized (7.0 cm) juveniles, a high density can also exacerbate size heterogeneity and cause death. Taking into account the welfare and yield of farmed seahorses, the present study suggests that the suitable stocking densities for 4.0, 7.0, and 9.0 cm juveniles in RASs are 0.6, 0.4, and 0.2 inds/L, respectively.

## 1. Introduction

Aquatic products have become the third-largest source of protein in the human diet after cereals and milk [1]. Due to the rapid decline in wild stock resources caused by overfishing and habitat degradation, aquaculture has replaced wild capture as the main means of supplying aquatic products [2]. There are generally two modes of aquaculture: the traditional extensive mode (e.g., outdoor pond farming) and the modern intensive mode (e.g., indoor factory farming) [3]. The latter can be subdivided into flow-through systems (FTSs) and recirculating aquaculture systems (RASs). FTSs usually pose a high risk of environmental pollution because their wastewater is directly discharged into the environment without treatment, while RASs are environmentally friendly and sustainable because their wastewater is treated and reused with almost zero discharge [4,5,6]. In the past decade, RASs have become the mainstream mode of aquaculture [3]. Although RASs are widely recommended, they also have limitations, with the most prominent being high factory and equipment investments and high running costs (mainly electricity) [7]. Therefore, this mode is selective in terms of the farming objects, generally requiring them to have high economic value. It is estimated that farming enterprises are likely to make profits only when the market price of the farming objects exceeds USD ten per kilogram [8,9]. Thus far, there are limited objects suitable for RASs, mainly comprising flatfish, salmon, and groupers [7].

The seahorse (*Hippocampus* genus, Syngnathidae family) is a new farming object that has only emerged in the past two decades [10,11]. It has high economic value and is mainly traded as traditional Chinese medicine, ornamental fish, and curios. A brightly colored live specimen is priced at over USD 60, and a dried specimen weighing two grams is sold for over USD 3 [12]. They also have a high market demand: over 37 million specimens are traded every year [13]. Therefore, seahorses may be a promising object suitable for RASs. However, to date, there are almost no reports on the large-scale farming of seahorses using RASs, and some key parameters, such as stocking density, are still unclear.

Stocking density is an important parameter in aquaculture [14]. If the density is too low, it will cause a waste of farming space and result in a low farming yield, while if the density is too high, it may have an impact on the growth and survival of the farmed organisms, which, in turn, may affect the farming yield [15]. In farming production, it is common for the farming yield to decrease rather than increase with an increase in the stocking density. The main reason for this decrease in yield is that high-density farming often produces a crowding effect, which has a series of adverse effects on farmed organisms, such as causing anorexia, stunted growth, inactive behavior, and sluggish reactions and even triggering various diseases [16,17,18]. Therefore, optimizing the stocking density of different farmed organisms based on their biological characteristics is a prerequisite for their commercial aquaculture.

The lined seahorse (*Hippocampus erectus*) is a seahorse species for which successful commercial aquaculture has been achieved. There is a large lined seahorse farming area in China, and its existing aquaculture model is an FTS [19,20]. In recent years, with the Chinese government’s control over the discharge standards and amounts of aquaculture wastewater, as well as farmers’ increasing awareness that the economic benefits of seahorse aquaculture are sufficient to cover the high costs of RASs, some farming enterprises have begun to attempt to cultivate lined seahorses in RASs. However, due to the unsuitable stocking density, their farming profits are not ideal. In the present study, we designed various density gradients in RASs and evaluated the effect on the cultivation of lined seahorse juveniles by analyzing their survival, growth, size homogeneity, and stress response. We employed plasma cortisol and brain serotonin (5-hydroxytryptamine, 5-HT) as indicators of stress levels; this is because, in teleosts, plasma cortisol has been proven to be a reliable indicator for indicating the stress response [21,22], and brain 5-HT has been reported to play a crucial role in behavioral and emotional control, as well as in neuroendocrine responses to stress [23,24]. Sustained serotonergic activation usually indicates that the fish is in chronic stress or in a stress-induced pathology (e.g., a depression-like state) [25,26,27]. The aim of the present study was to determine the optimal stocking density for lined seahorse juveniles at different growth stages in RASs.

## 2. Materials and Methods

### 2.1. Ethical Approval

Ethical approval for this study was granted by the Committee on the Ethics of Animal Experiments of the Chinese Academy of Fishery Sciences, with approval reference number CAFS-EC-2023-086. All procedures were conducted in compliance with the recommendations in the Guide for the Management and Use of the Experimental Animals of China Science and Technology Commission.

### 2.2. Experimental Seahorses

At present, in China, the general operations when using an RAS to cultivate the lined seahorse are as follows: The farming enterprise first purchases one-month-old juveniles with a body height (the straight-line distance from the tip of the head coronet to the tip of the uncurled tail) of about 4.0 cm from a hatchery, and then it cultivates them in an RAS for three months (at this point, the body height of the juveniles reaches about 11.0 cm) before they are put on the market. During these three months, as the juveniles gradually grow, the farming enterprise dilutes the stocking density after each month of cultivation. Therefore, using one month of cultivation as the time point, the present study focused on three different sizes of juveniles, namely, one-month-old juveniles (just entered the system), two-month-old juveniles (cultivated in the system for one month, with a body height of about 7.0 cm), and three-month-old juveniles (cultivated in the system for two months, with a body height of about 9.0 cm), to investigate their optimal stocking densities.

The present study was carried out at Wenchang Innovation Research Center, Fengjiawan Modern Fishery Industry Park, Hainan Province, China, from September 2023 to February 2024. The center houses hundreds of thousands of artificially bred lined seahorses of various sizes and several sets of standardized RASs. The experimental seahorses (i.e., the lined seahorse juveniles with initial body heights of 4.0, 7.0, and 9.0 cm) and experimental systems (i.e., RAS) used in the present study were provided for free by the center.

### 2.3. Experimental System

**Figure 1:**
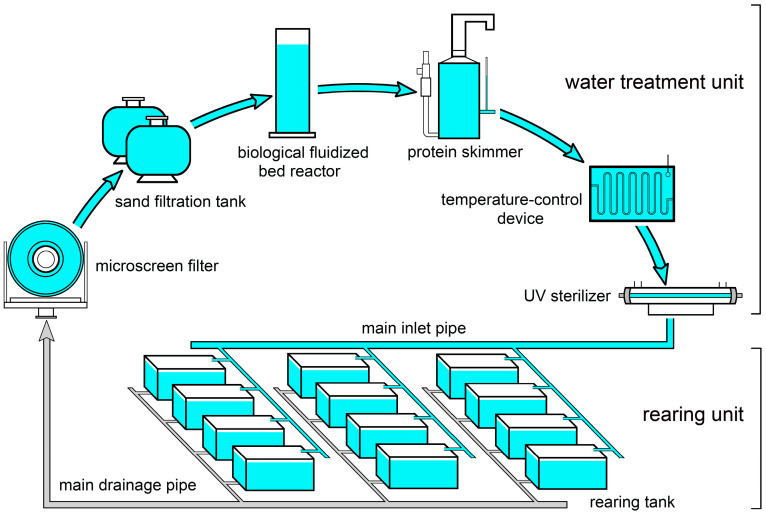
Frame of the recirculating aquaculture system used in the present study.

The RAS consisted of a water treatment unit and a rearing unit. The water treatment unit included a microscreen filter, two sand filtration tanks filled with quartz sand with a particle diameter of 4–8 mm, a biological fluidized bed reactor, a protein skimmer, a temperature control device, and a UV sterilizer (Figure 1). The rearing unit consisted of 24 rectangular fiberglass tanks. The length, width, and height of each tank were 140 cm, 90 cm, and 90 cm, respectively, with a water capacity of 1000 L. Each tank was equipped with a water inlet and outlet positioned at the water surface. Each tank contained several plastic mesh slices for the seahorses to attach to. The tailwater from each tank flowed into the same large pipe (i.e., the main drainage pipe), then flowed back to the water treatment unit, passed through the water treatment components in sequence, and finally flowed into another large pipe (the main inlet pipe) for distribution to each tank.

### 2.4. Water and Rearing Conditions

The temperature of the rearing water was 25–26 °C, and it was controlled by the temperature control system. The salinity, pH, and dissolved oxygen of the rearing water were 28–29‰, 8.0–8.2, and 6.5–8.0 mg/L, respectively. The ammonia and nitrite were 0.002–0.003 mg/L and 0.01–0.10 mg/L, respectively. The light intensity was 1500–2000 lux, and the photoperiod was 12 h L/12 h D. The inlet water flow rate of each tank was 4.0–5.0 L/min.

### 2.5. Stocking Density

**Figure 2:**
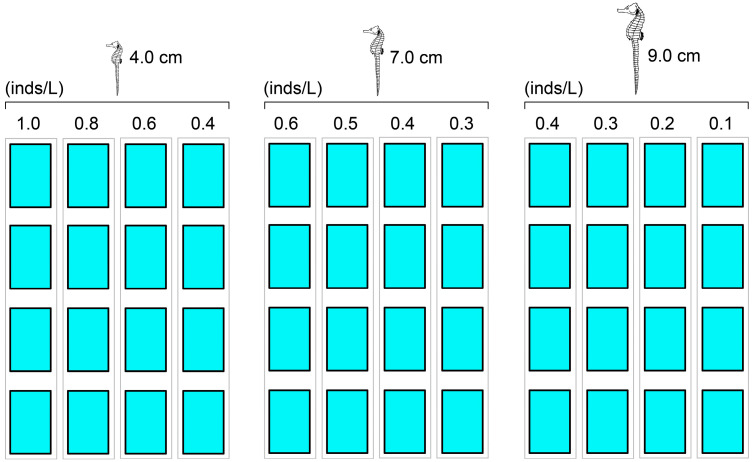
Panoramic view of the experimental design with test stocking densities.

The test density gradients of the three experimental seahorses, i.e., the 4.0, 7.0, and 9.0 cm juveniles, were 1.0, 0.8, 0.6, and 0.4 inds/L; 0.6, 0.5, 0.4, and 0.3 inds/L; and 0.4, 0.3, 0.2, and 0.1 inds/L, respectively. Each gradient had four replicates (Figure 2).

The juveniles with an initial body height of 4.0 cm (i.e., the one-month-old juveniles; the actual measurement values of the body height and wet weight were 4.02 ± 0.14 cm and 0.210 ± 0.031 g) were placed in the RAS rearing tanks according to their corresponding densities. They were fed twice a day (08:00 and 14:00) ad libitum with live copepods for 30 days. The copepods were collected daily in the early morning from outdoor shrimp farming ponds and disinfected with 5 ppm povidone-iodine for one hour before feeding. To prevent the copepods from escaping from the water outlet, a lid made of a plastic fence mesh, which was wrapped in a 120-mesh screen (mesh size of 125 μm), was used to cover the water outlet. Three hours after each feeding, the feces and dead copepods in the tanks were siphoned out, and the lids were brushed clean. Dead seahorses, if any, were picked up and recorded during daily inspections.

The juveniles with initial body heights of 7.0 cm (i.e., the two-month-old juveniles; the actual measurement values of the body height and wet weight were 6.98 ± 0.14 cm and 1.153 ± 0.099 g) and 9.0 cm (i.e., the three-month-old juveniles; the actual measurement values of the body height and wet weight were 8.92 ± 0.14 cm and 3.030 ± 0.175 g) were placed in the RAS rearing tanks according to their corresponding densities. They were fed twice a day (08:00 and 15:00) ad libitum with frozen Mysis for 30 days. The reason why the juveniles’ feed was changed from copepods to Mysis is that one-month-old juveniles cannot swallow the entire Mysis due to their small snout, so they prefer copepods during this growth stage; in contrast, two-month-old juveniles are able to swallow the entire Mysis, so from this stage of growth, they prefer Mysis. Frozen Mysis was purchased from Hikari™, Kyorin Industry (Shenzhen) Co., Ltd., Guangzhou, China. It was first thawed, then rinsed with clean water, drained, and finally fed to the juveniles. To allow the shrimp fragments on the water surface to flow out as much as possible from the water outlet, the screen used to wrap the outlet lid was changed to a 10-mesh screen (mesh size of 2 mm). Two hours after each feeding, the feces and uneaten shrimp were siphoned out, and the lids were brushed clean. Dead seahorses, if any, were picked up and recorded during daily inspections.

### 2.6. Survival and Growth Measurements

After 30 days of feeding, the total number of dead seahorses (N_d_) in each replicate of each stocking density was counted. The survival rate (SR) was calculated as

SR = 100 (N_0_ − N_d_)/N_0_, (1)

where N_0_ is the initial number of live seahorses.

After 30 days of feeding, 40 seahorses were randomly sampled from each replicate of each stocking density to measure their final body height (*H*_f_) and final wet weight (*W*_f_) for the calculation of the specific growth rate (SGR) and Fulton’s condition factor (*K*).

SGR = 100 (ln *W*_f_ − ln *W*_i_)/time, (2)

*K* = 100 (*W*_f_/*H*_f_^3^), (3)

where *W*_i_ is the initial wet weight.

The seahorse body height, referring to the straight-line distance from the tip of the head coronet to the tip of the uncurled tail, was measured with a steel ruler with a precision of 0.1 cm (i.e., 1 mm), and the seahorse wet weight was measured using a Mettler Toledo balance with a precision of 0.001 g (i.e., 1 mg).

### 2.7. Size Heterogeneity Analysis

In production practice, it is often found that juvenile lined seahorses of the same age in the same rearing tank tend to have uneven sizes, with some of them being particularly large and some of them being particularly small. We speculate that this size heterogeneity may be related to the stocking density. To test this speculation, we employed the coefficient of variation (CV) to analyze the size heterogeneity of the juveniles at different densities.

CV = 100 [standard deviation (SD)/mean].(4)

### 2.8. Stress Level Determinations

After 30 days of feeding, another six seahorses were randomly sampled from each replicate of each stocking density and placed one by one in seawater containing 0.02% MS-222 for anesthesia for three minutes. Blood was collected using the tail-cutting method. Specifically, a section of the tail of the anesthetized seahorse was cut off; then, the remaining tail was immediately inserted into a 1.5 mL centrifuge tube containing 0.6 mL of anticoagulant (citric acid 0.48 g, sodium citrate 1.32 g, glucose 1.47 g, and distilled water 100 mL) and dipped into the anticoagulant. Blood spontaneously flowed out of the caudal artery and mixed with the anticoagulant. Due to the small volume of blood in each seahorse, the blood from six seahorses was pooled in the same centrifuge tube. The mixture of blood and anticoagulant was left to stand for 10 min at 4 °C and then centrifuged at 840× *g* for 10 min at 4 °C (Sigma 3K18, Sigma, Kanagawa, Japan) to collect the supernatant (i.e., plasma) [28]. The plasma was stored at −80 °C for a later analysis of cortisol.

After blood collection, the head coronet of the amputated seahorse was cut open, and the brain tissue was quickly collected within 30 s. Due to the small amount of brain tissue in each seahorse, the brain tissues from six seahorses were mixed in the same sampling tube. The brain tissues were homogenized in ice-cold phosphate-buffered saline (PBS, sodium chloride 0.8 g, potassium chloride 0.02 g, disodium hydrogen phosphate 0.29 g, potassium dihydrogen phosphate 0.02 g, and distilled water 100 mL) at pH 7.2 using a Potter–Elvehjem homogenizer and then centrifuged at 840× *g* for 10 min at 4 °C to collect the supernatant [27]. The supernatant was stored at −80 °C for a later analysis of 5-HT.

Cortisol and 5-HT were measured with commercial ELISA kits (MEIMIAN, Jiangsu Meimian Industrial Co., Ltd., Jiangsu, China) suitable for fish following the manufacturer’s instructions. The protein concentrations in the plasma and brain tissue supernatant were measured using Bradford’s method [29]. The unit of cortisol is expressed as the amount contained in each milligram of plasma protein, and the unit of 5-HT is expressed as the amount contained in each milligram of brain tissue protein.

### 2.9. Statistical Analysis

All data are expressed as the mean ± standard deviation (SD) and were analyzed using Origin PRO graphing and analysis software (Version 9.2.214). The normality of all data was evaluated using the Shapiro–Wilk test [30], and the homogeneity of the variances was assessed using Levene’s test. If the variance was homogeneous, a one-way analysis of variance (ANOVA) was used to analyze the differences among the four stocking densities; if the variance was not homogeneous, the Kruskal–Wallis *H* test (a non-parametric test) [30] was used to analyze the differences among the four stocking densities. If the one-way ANOVA result showed a significant difference (*p* < 0.05) among the four stocking densities, then Tukey’s multiple comparison was applied, and if the Kruskal–Wallis *H* test result showed a significant difference, then Dunn’s multiple comparison was applied.

## 3. Results

### 3.1. Juveniles with a Body Height of 4.0 cm

#### 3.1.1. Survival and Growth

The survival and growth of the juveniles with an initial body height of 4.0 cm at the end of the trial are shown in Table 1. The survival rate results showed significant differences among the four density groups (*F*_3, 15_ = 114.21, *p* < 0.001). Tukey’s multiple comparisons showed that there was no significant difference among the 0.8, 0.6, and 0.4 inds/L groups, and their values were 90.88 ± 2.06%, 92.79 ± 1.14%, and 94.00 ± 1.32%, respectively, while the value of the 1.0 inds/L group was only 71.18 ± 2.99%, which was significantly lower than that of the other three groups.

The results of the final body height (*H*_3_ = 197.04, *p* < 0.001), final wet weight (*H*_3_ = 190.00, *p* < 0.001), and SGR (*H*_3_ = 190.00, *p* < 0.001) also showed significant differences among the four density groups. Dunn’s multiple comparisons showed that the low-density groups (i.e., 0.6 and 0.4 inds/L) had better growth performance, and their final body height, final wet weight, and SGR were 1.13-, 1.43-, and 1.33-fold higher than those in the high-density groups (i.e., 1.0 inds/L and 0.8 inds/L), respectively. The *K* (Fulton’s condition factor) results also showed significant differences among the four density groups (*F*_3, 639_ = 4.86, *p* = 0.002). Tukey’s multiple comparisons showed that the 0.4 inds/L group had a significantly higher value, and the other three groups showed no significant difference.

#### 3.1.2. Size Heterogeneity

**Figure 3:**
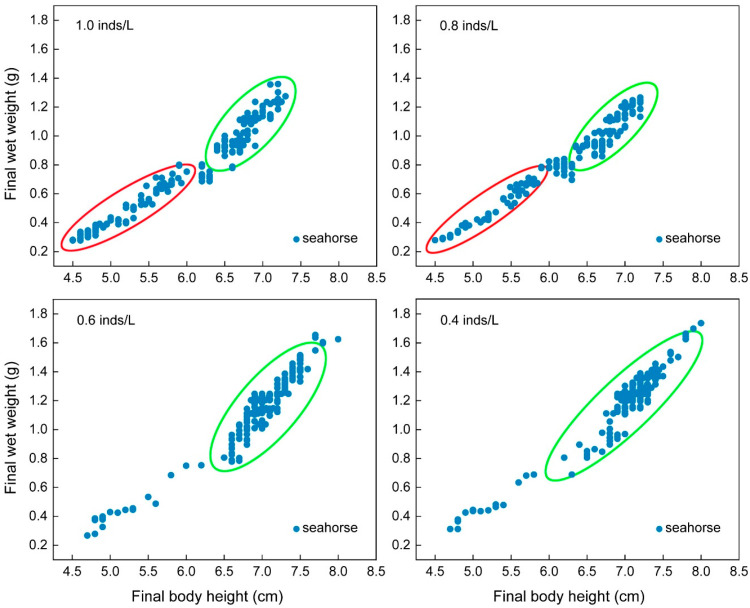
The final body height and wet weight of the juveniles with an initial body height of 4.0 cm (real body height: 4.02 ± 0.14 cm; real wet weight: 0.210 ± 0.031 g) after one month of rearing. Each solid blue dot represents a seahorse juvenile. The red and green circles represent populations of different body sizes.

The CVs of the final body height (*F*_3, 15_ = 36.46, *p* < 0.001) and final wet weight (*F*_3, 15_ = 30.82, *p* < 0.001) showed significant differences among the four density groups. Tukey’s multiple comparisons showed that the variation was the highest in the 1.0 inds/L group, followed by the 0.8 inds/L group, and it was the lowest in the 0.6 and 0.4 inds/L groups. The final body height and final wet weight CVs of the seahorses reared at 1.0 inds/L were 1.55- and 1.66-fold higher than those of the seahorses reared at 0.4 and 0.6 inds/L, respectively (Table 1). The final body height and final wet weight of each seahorse were examined in scatter plots. The scatter plots of the high-density groups (i.e., 1.0 and 0.8 inds/L) were more scattered and could be roughly divided into two size populations (green and red circles), while the scatter plots of the low-density groups (i.e., 0.6 and 0.4 inds/L) were more concentrated and contained one size population (green circle) (Figure 3).

#### 3.1.3. Stress Level

**Figure 4:**
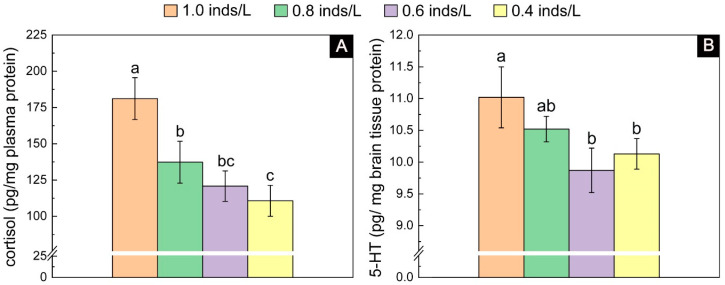
The plasma cortisol (**A**) and brain 5-HT (**B**) contents in the juveniles with an initial body height of 4.0 cm (real body height: 4.02 ± 0.14 cm; real wet weight: 0.210 ± 0.031 g) after one month of rearing. The lowercase letters (a, b, and c) on each density bar chart indicate the results of multiple comparisons, and the absence of shared letters between two densities indicates significant differences between them.

The cortisol results showed significant differences among the four density groups (*F*_3, 15_ = 24.20, *p* < 0.001). Tukey’s multiple comparisons showed that the 1.0 inds/L group had the highest level of cortisol, which was significantly higher than that of the other three groups. The lowest level of cortisol was present in the low-density groups (i.e., 0.6 and 0.4 inds/L), whose value was 36.09% lower than that of the 1.0 inds/L group (Figure 4A). Similarly, the 5-HT results also showed significant differences among the four density groups (*F*_3, 15_ = 8.73, *p* = 0.002). Tukey’s multiple comparisons showed that the 1.0 inds/L group had the highest level, while the 0.6 and 0.4 inds/L groups had the lowest level, and the highest level was 1.10 times higher than the lowest level (Figure 4B).

### 3.2. Juveniles with a Body Height of 7.0 cm

#### 3.2.1. Survival and Growth

The survival and growth of the juveniles with an initial body height of 7.0 cm at the end of the trial are shown in Table 2. The survival rate results showed significant differences among the four density groups (*F*_3, 15_ = 16.46, *p* < 0.001). Tukey’s multiple comparisons showed that there was no significant difference among the 0.5, 0.4, and 0.3 inds/L groups, and their values were 83.40 ± 2.57%, 84.63 ± 2.10%, and 87.83 ± 2.53%, respectively, while the value of the 0.6 inds/L group was only 76.38 ± 2.30%, which was significantly lower than that of the other three groups.

The final body height results showed significant differences among the four density groups (*H*_3_ = 104.38, *p* < 0.001). Dunn’s multiple comparisons showed that the 0.4 and 0.3 inds/L groups had significantly higher values, and there was no significant difference between the values of the 0.6 inds/L and 0.5 inds/L groups. The value in the low-density groups (i.e., 0.4 and 0.3 inds/L) was 1.05-fold higher than that in the high-density groups (i.e., 0.6 and 0.5 inds/L). The results of the final wet weight (*H*_3_ = 132.89, *p* < 0.001), SGR (*H*_3_ = 132.89, *p* < 0.001), and *K* (*H*_3_ = 78.72, *p* < 0.001) all showed significant differences among the four density groups. Dunn’s multiple comparisons showed that the low-density groups (i.e., 0.4 and 0.3 inds/L) had the best growth performance, followed by the 0.5 inds/L group, and the 0.6 inds/L group had the worst. The final wet weight, SGR, and *K* in the best groups were 1.27-, 1.38-, and 1.08-fold higher than those in the worst group, respectively.

#### 3.2.2. Size Heterogeneity

**Figure 5:**
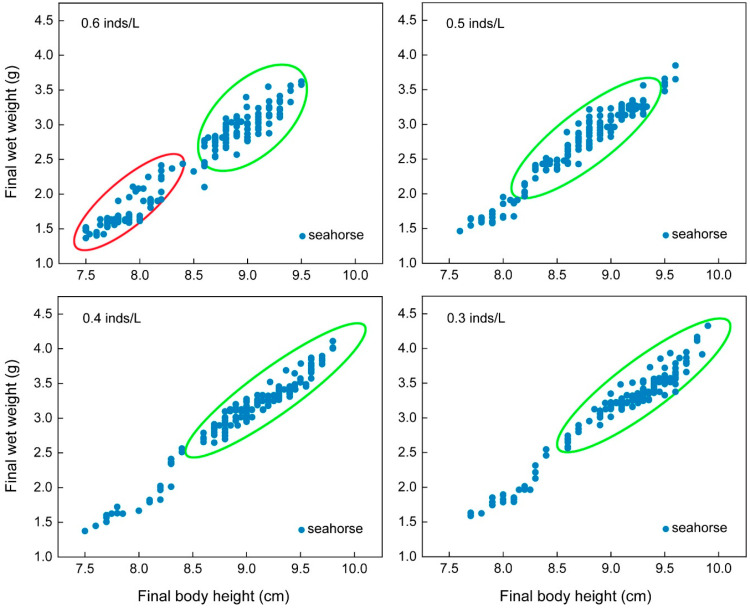
The final body height and wet weight of the juveniles with an initial body height of 7.0 cm (real body height: 6.98 ± 0.14 cm; real wet weight: 1.153 ± 0.099 g) after one month of rearing. Each solid blue dot represents a seahorse juvenile. The red and green circles represent populations of different body sizes.

The CVs of the final body height (*F*_3, 15_ = 13.31, *p* < 0.001) and final wet weight (*F*_3, 15_ = 37.00, *p* < 0.001) showed significant differences among the four density groups (Table 2). Tukey’s multiple comparisons showed that the 0.6 inds/L group had the highest variations, which were significantly higher than those of the other three groups, while there was no significant difference among the other three groups. The final body height and final wet weight CVs of the 0.6 inds/L group were 1.25- and 1.41-fold higher than those of the other three groups, respectively. The final body height and final wet weight of each seahorse were examined in scatter plots. The scatter plot of the 0.6 inds/L group was more scattered and could be roughly divided into two size populations (green and red circles), while the scatter plots of the other three groups were more concentrated and contained one size population (green circle) (Figure 5).

#### 3.2.3. Stress Level

**Figure 6:**
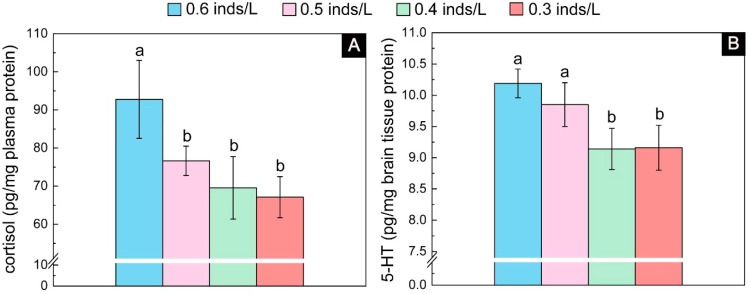
The plasma cortisol (**A**) and brain 5-HT (**B**) contents in the juveniles with an initial body height of 7.0 cm (real body height: 4.02 ± 0.14 cm; real wet weight: 0.210 ± 0.031 g) after one month of rearing. The lowercase letters (a and b) on each density bar chart indicate the results of multiple comparisons, and the absence of shared letters between two densities indicates significant differences between them.

The cortisol results showed significant differences among the four density groups (*F*_3, 15_ = 9.89, *p* = 0.001). Tukey’s multiple comparisons showed that the 0.6 inds/L group had the highest value, which was significantly higher than that of the other three groups, whereas there was no significant difference among the other three groups (Figure 6A). The value of the 0.6 inds/L group was 1.30 times higher than the mean of the other three groups. Similarly, the 5-HT results also showed significant differences among the four density groups (*F*_3, 15_ = 10.26, *p* = 0.001). High values were found in the high-density groups (i.e., 0.6 and 0.5 inds/L), and low values were found in the low-density groups (i.e., 0.4 and 0.3 inds/L), and the high values were 1.09 times higher than the low values (Figure 6B).

### 3.3. Juveniles with a Body Height of 9.0 cm

#### 3.3.1. Survival and Growth

The survival and growth of the juveniles with an initial body height of 9.0 cm at the end of the trial are shown in Table 3. The survival rate results showed no significant difference among the four density groups (*F*_3, 15_ = 1.26, *p* = 0.331), and their mean value was 96.61 ± 1.13%.

The results of the final body height (*F*_3, 639_ = 22.08, *p* < 0.001), final wet weight (*H*_3_ = 81.16, *p* < 0.001), and SGR (*F*_3, 639_ = 22.76, *p* < 0.001) showed significant differences among the four density groups. Multiple comparisons showed that the low-density groups (i.e., 0.1 and 0.2 inds/L) had the best growth performance, followed by the 0.3 inds/L group, and the 0.4 inds/L group had the worst. The final body height, final wet weight, and SGR in the best groups were 1.04-, 1.15-, and 1.24-fold higher than those in the worst group, respectively. The *K* results also showed significant differences among the four density groups (*F*_3, 639_ = 15.20, *p* < 0.001). Tukey’s multiple comparisons showed that the 0.1 and 0.2 inds/L groups had significantly higher values, and there was no significant difference between the values of the 0.4 inds/L and 0.3 inds/L groups.

#### 3.3.2. Size Heterogeneity

**Figure 7:**
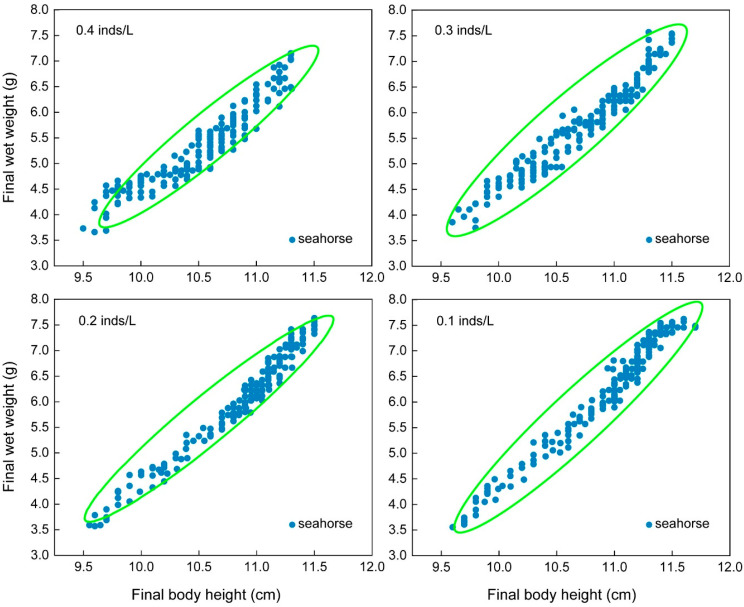
The final body height and wet weight of the juveniles with an initial body height of 9.0 cm (real body height: 8.92 ± 0.14 cm; real wet weight: 3.030 ± 0.175 g) after one month of rearing. Each solid blue dot represents a seahorse juvenile. The green circle represents a population of one body size.

The CVs of the final body height (*F*_3, 15_ = 0.21, *p* = 0.888) and final wet weight (*F*_3, 15_ = 1.59, *p* = 0.243) showed no significant differences among the four density groups (Table 3). The final body height and final wet weight of each seahorse were examined in scatter plots. The scatter plots of the four density groups all roughly contained one size population (green circle) (Figure 7).

#### 3.3.3. Stress Level

**Figure 8:**
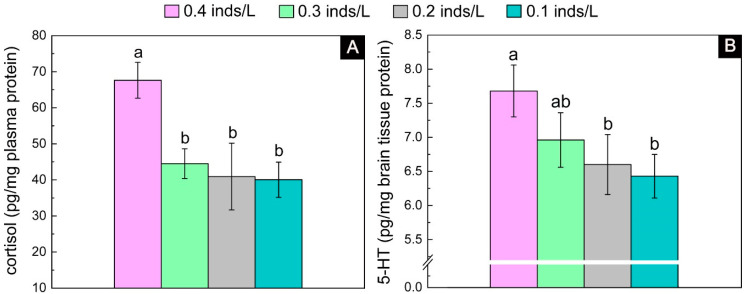
The plasma cortisol (**A**) and brain 5-HT (**B**) contents in the juveniles with an initial body height of 9.0 cm (real body height: 8.92 ± 0.14 cm; real wet weight: 3.030 ± 0.175 g) after one month of rearing. The lowercase letters (a and b) on each density bar chart indicate the results of multiple comparisons, and the absence of shared letters between two densities indicates significant differences between them.

The cortisol results showed significant differences among the four density groups (*F*_3, 15_ = 17.92, *p* < 0.001). Tukey’s multiple comparisons showed that the 0.4 inds/L group had a significantly higher value, and the other three groups showed no significant difference. The value of the 0.4 inds/L group was 1.62 times higher than the mean of the other three groups (Figure 8A). Similarly, the 5-HT results also showed significant differences among the four density groups (*F*_3, 15_ = 8.19, *p* = 0.003). Tukey’s multiple comparisons showed that the 0.4 inds/L group had the highest value, followed by the 0.3 inds/L group, and the 0.2 and 0.1 inds/L groups had the lowest value; furthermore, the highest value was 1.18 times higher than the lowest value (Figure 8B).

## 4. Discussion

The present study demonstrated that the stocking density has a significant impact on the survival, growth, size homogeneity, and physiological stress of the juvenile lined seahorse (*H. erectus*). Specifically, a high density typically leads to a reduced survival rate, slowed growth, and an increased stress response. A high density also exacerbates the size heterogeneity of small- and medium-sized juveniles (e.g., juveniles with initial body heights of 4.0 cm and 7.0 cm). Taking into account the welfare and yield per cubic meter of water of farmed seahorses, the present study suggests that the suitable stocking densities for lined seahorses with initial body heights of 4.0 cm, 7.0 cm, and 9.0 cm in RASs are 0.6 inds/L, 0.4 inds/L, and 0.2 inds/L, respectively.

A high stocking density has been demonstrated to inhibit growth and even cause death in many farmed organisms [15,17,18,31]. The results of the present study show that this adverse effect also occurs in the lined seahorse, and, regardless of the size of the juveniles, their growth rates decreased with an increasing stocking density. In the case of sufficient food and almost identical rearing facilities and water (the present study used recirculating water), the growth rate in high-density cultivation was lower than that in low-density cultivation, which was likely related to the crowding stress caused by a high density. In teleosts, plasma cortisol is a reliable indicator of stress, and brain 5-HT is also gradually being recognized as a neuroendocrine indicator related to emotions and stress; high levels of these usually indicate that the organism is in a stressed state, even in a depression-like state [21,22,27]. In the present study, the stress response data showed that, regardless of the size of the juveniles, if they were under a high density, their plasma cortisol and brain 5-HT were at a high level, proving that the juveniles under a high density were in a state of crowding stress. The most direct effect of crowding stress is that it drives the farmed organisms to allocate more energy to compete for the space that they need for survival, resulting in a decrease in the energy used for growth or immunity [18,32]. In addition, crowding stress can also trigger a series of indirect effects, such as decreased appetite, reduced food conversion efficiency, inflammatory reactions, and weakened antioxidant capacity [18,33,34,35]. These direct and indirect effects are largely the main causes of slow growth.

In the present study, the stress response data also showed that the cortisol and 5-HT contents decreased with the growth of the seahorses. A similar result was also observed in Atlantic salmon (*Salmo salar*): the plasma cortisol and brain 5-HT contents in fish sampled in the second month (length: 24.7 ± 0.6 cm, weight: 176 ± 13.1 g) were higher than those in fish sampled in the fifth month (length: 38.2 ± 1.2 cm, weight: 721 ± 81.4 g) [27]. The decreases in the cortisol (pg/mg plasma protein) and 5-HT contents (pg/mg brain tissue protein) may be due to a decrease in their secretion (i.e., numerator decrease) or an increase in other protein components in plasma and brain tissue (denominator increase) with the growth of farmed organisms. The lower levels of cortisol and 5-HT in larger fish may suggest that they have a greater tolerance or adaptability to stress than smaller fish, as inferred by Stevens et al. [36], who stated that small fish are likely to be more vulnerable and sensitive than large fish when exposed to stress. In support of this, the present study found that, when suffering from crowding stress, small-sized seahorses (an initial body height of 4.0 cm at a 1.0 inds/L density) showed a number of deaths, with a survival rate of about 71%, while the large-sized seahorses (an initial body height of 9.0 cm at a 0.4 inds/L density) showed almost no deaths, with a survival rate of about 96%, which proves that large fish have a stronger resistance to stress than small fish. In addition, we also found that some deaths occurred in juveniles with an initial body height of 7.0 cm, regardless of the density, and their survival rates were all below 90%. These deaths may be related to the change in food, as the seahorse juveniles with an initial body height of 7.0 cm began to switch from feeding on live copepods to frozen Mysis. During this process, a small portion of juveniles died due to their inability to adapt to frozen food [19,37]. When the seahorses were fully adapted to the frozen food, there were no more deaths.

The results of the present study also show that the final body height and final wet weight CVs for the small- and medium-sized juveniles in high-density cultivation (e.g., 4.0 cm juveniles at 1.0 and 0.8 inds/L and 7.0 cm juveniles at 0.6 inds/L) were higher than those in low-density cultivation, indicating that high-density cultivation exacerbates the size heterogeneity of small- and medium-sized juveniles. This size heterogeneity may be related to the social hierarchy of the lined seahorse. It is generally believed that the higher the population density, the more likely it is that a social hierarchy forms [38]. In high-density cultivation, there is an intense intraspecific competition for food and space between fish. Due to differences in body size, personality traits, and social experience, their competitive abilities also vary, which determines their different social ranks in competition [39,40,41]. Dominant fish usually have a larger body size and food intake, stronger food and space acquisition abilities, and lower predation risks and stress responses, which contribute to their rapid growth. On the contrary, subordinate fish usually have a smaller body size, a lower food intake, and weaker food and space acquisition abilities, and they are subjected to stresses such as attack and intimidation from dominant fish, which lead to their growth retardation [41,42,43]. Therefore, the social hierarchy is likely to be the reason for the large deviation of fish in high-density cultivation. Unlike the small- and medium-sized juveniles, the size heterogeneity of the large-sized juveniles (initial body height of 9.0 cm) did not exacerbate in high-density cultivation. A possible reason for this is that, among the large-sized juveniles, dominant fish began to allocate a portion of energy to gonadal and brood pouch development, leading to a slowdown in their growth [44,45]. The growth slowdown of dominant fish led to a weakening of their body size advantage, thereby improving the social rank of subordinate fish. The abilities of subordinate fish to acquire food and space were strengthened, and their growth also accelerated accordingly [38,46]. Thus, the growth slowdown of dominant fish and the growth acceleration of subordinate fish may be the reason why the size heterogeneity of the large-sized juveniles was not exacerbated in high-density cultivation.

## 5. Conclusions

In the present study, we demonstrated that the stocking density has a significant impact on the survival, growth, size homogeneity, and physiological stress of the juvenile lined seahorse (*H. erectus*). A high density can lead to reduced survivorship, slowed growth, an increased stress response, and an exacerbated size heterogeneity. Therefore, the recommended stocking densities for 4.0 cm, 7.0 cm, and 9.0 cm juvenile lined seahorses in RASs are 0.6 inds/L, 0.4 inds/L, and 0.2 inds/L, respectively.

## Figures and Tables

**Table 1:** The survival rate and growth parameters (mean ± SD) of the juveniles with an initial body height of 4.0 cm after one month of rearing.

	Stocking Density (inds/L)				*p*-Value
	1.0	0.8	0.6	0.4	
Survival rate (%)	71.18 ± 2.99 ^b^	90.88 ± 2.06 ^a^	92.79 ± 1.14 ^a^	94.00 ± 1.32 ^a^	<0.001
Final BH (cm)	6.03 ± 0.89 ^b^	6.16 ± 0.79 ^b^	6.87 ± 0.70 ^a^	6.95 ± 0.65 ^a^	<0.001
Final WW (g)	0.773 ± 0.325 ^b^	0.812 ± 0.289 ^b^	1.107 ± 0.304 ^a^	1.166 ± 0.286 ^a^	<0.001
SGR	3.99 ± 1.61 ^b^	4.24 ± 1.44 ^b^	5.36 ± 1.24 ^a^	5.56 ± 1.11 ^a^	<0.001
*K*	0.329 ± 0.025 ^b^	0.330 ± 0.022 ^b^	0.331 ± 0.024 ^b^	0.338 ± 0.022 ^a^	0.002
CV of final BH	14.84 ± 0.70 ^a^	12.94 ± 0.91 ^b^	9.75 ± 0.99 ^c^	9.38 ± 0.85 ^c^	<0.001
CV of final WW	42.24 ± 3.21 ^a^	35.83 ± 2.72 ^b^	26.25 ± 3.13 ^c^	24.57 ± 2.91 ^c^	<0.001

BH: body height; WW: wet weight; SGR: specific growth rate; *K*: Fulton’s condition factor; CV: coefficient of variation. The superscript lowercase letters (a, b, and c) after each value in the same row indicate the results of multiple comparisons, and the absence of shared letters between two values indicates significant differences between them.

**Table 2:** The survival rate and growth parameters (mean ± SD) of the juveniles with an initial body height of 7.0 cm after one month of rearing.

	Stocking Density (inds/L)				*p*-Value
	0.6	0.5	0.4	0.3	
Survival rate (%)	76.38 ± 2.30 ^b^	83.40 ± 2.57 ^a^	84.63 ± 2.10 ^a^	87.83 ± 2.53 ^a^	<0.001
Final BH (cm)	8.49 ± 0.60 ^b^	8.69 ± 0.48 ^b^	8.98 ± 0.50 ^a^	9.04 ± 0.55 ^a^	<0.001
Final WW (g)	2.409 ± 0.679 ^c^	2.681 ± 0.569 ^b^	3.041 ± 0.575 ^a^	3.089 ± 0.624 ^a^	<0.001
SGR	2.31 ± 1.00 ^c^	2.73 ± 0.78 ^b^	3.16 ± 0.75 ^a^	3.20 ± 0.81 ^a^	<0.001
*K*	0.382 ± 0.038 ^c^	0.401 ± 0.032 ^b^	0.414 ± 0.028 ^a^	0.410 ± 0.027 ^a^	<0.001
CV of final BH	7.08 ± 0.30 ^a^	5.50 ± 0.50 ^b^	5.52 ± 0.48 ^b^	6.00 ± 0.30 ^b^	<0.001
CV of final WW	28.36 ± 1.36 ^a^	21.22 ± 1.88 ^b^	18.81 ± 1.13 ^b^	20.11 ± 1.10 ^b^	<0.001

BH: body height; WW: wet weight; SGR: specific growth rate; *K*: Fulton’s condition factor; CV: coefficient of variation. The superscript lowercase letters (a, b, and c) after each value in the same row indicate the results of multiple comparisons, and the absence of shared letters between two values indicates significant differences between them.

**Table 3:** The survival rate and growth parameters (mean ± SD) of the juveniles with an initial body height of 9.0 cm after one month of rearing.

	Stocking Density (inds/L)				*p*-Value
	0.4	0.3	0.2	0.1	
Survival rate (%)	95.94 ± 0.85	96.25 ± 0.83	97.25 ± 1.19	97.00 ± 1.41	0.331
Final BH (cm)	10.53 ± 0.47 ^c^	10.71 ± 0.50 ^b^	10.87 ± 0.49 ^a^	10.93 ± 0.49 ^a^	<0.001
Final WW (g)	5.352 ± 0.792 ^c^	5.713 ± 0.941 ^b^	6.089 ± 1.014 ^a^	6.207 ± 1.035 ^a^	<0.001
SGR	1.86 ± 0.49 ^c^	2.07 ± 0.56 ^b^	2.27 ± 0.61 ^a^	2.34 ± 0.62 ^a^	<0.001
*K*	0.455 ± 0.021 ^b^	0.460 ± 0.019 ^b^	0.468 ± 0.023 ^a^	0.470 ± 0.024 ^a^	<0.001
CV of final BH	4.45 ± 0.42	4.66 ± 0.35	4.57 ± 0.31	4.53 ± 0.46	0.888
CV of final WW	14.80 ± 0.62	16.53 ± 1.42	16.66 ± 1.43	16.72 ± 2.02	0.243

BH: body height; WW: wet weight; SGR: specific growth rate; *K*: Fulton’s condition factor; CV: coefficient of variation. The superscript lowercase letters (a, b, and c) after each value in the same row indicate the results of multiple comparisons, and the absence of shared letters between two values indicates significant differences between them.

## Data Availability

The original data presented in the study are included in the article; further inquiries can be directed to the corresponding author.
